# Enhanced Room-Temperature Optoelectronic NO_2_ Sensing Performance of Ultrathin Non-Layered Indium Oxysulfide via In Situ Sulfurization

**DOI:** 10.3390/s26020670

**Published:** 2026-01-19

**Authors:** Yinfen Cheng, Nianzhong Ma, Zhong Li, Dengwen Hu, Zhentao Ji, Lieqi Liu, Rui Ou, Zhikang Shen, Jianzhen Ou

**Affiliations:** 1 Institute for Advanced Study, Chengdu University, Chengdu 610106, China; 2 Key Laboratory of Advanced Technologies of Materials, Ministry of Education, School of Materials Science and Engineering, Southwest Jiaotong University, Chengdu 610031, China; 3 School of Engineering, Royal Melbourne Institute of Technology University, Melbourne 3000, Australia; 4 College of Engineering and Technology, Southwest University, Chongqing 400715, China

**Keywords:** indium oxysulfide, NO_2_ gas sensing, room temperature, sulfurization, liquid metal printing process, two dimension

## Abstract

The detection of trace nitrogen dioxide (NO_2_) is critical for environmental monitoring and industrial safety. Among various sensing technologies, chemiresistive sensors based on semiconducting metal oxides are prominent due to their high sensitivity and fast response. However, their application is hindered by inherent limitations, including low selectivity and elevated operating temperatures, which increase power consumption. Two-dimensional metal oxysulfides have recently attracted attention as room-temperature sensing materials due to their unique electronic properties and fully reversible sensing performance. Meanwhile, their combination with optoelectronic gas sensing has emerged as a promising solution, combining higher efficiency with minimal energy requirements. In this work, we introduce non-layered 2D indium oxysulfide (In_2_S_x_O_3−x_) synthesized via a two-step process: liquid metal printing of indium followed by thermal annealing of the resulting In_2_O_3_ in a H_2_S atmosphere at 300 °C. The synthesized material is characterized by a micrometer-scale lateral dimension with 6.3 nm thickness and remaining n-type semiconducting behavior with a bandgap of 2.53 eV. It demonstrates a significant response factor of 1.2 toward 10 ppm NO_2_ under blue light illumination at room temperature. The sensor exhibits a linear response across a low concentration range of 0.1 to 10 ppm, alongside greatly improved reversibility, selectivity, and sensitivity. This study successfully optimizes the application of 2D metal oxysulfide and presents its potential for the development of energy-efficient NO_2_ sensing systems.

## 1. Introduction

With rapid industrialization, the threat posed by air pollution to global health has grown significantly in recent decades. Nitrogen dioxide (NO_2_), one of the most hazardous gases, is primarily emitted from industrial processes and vehicle exhaust [1,2]. NO_2_ not only causes acid rain and photochemical smog but also triggers serious health conditions including emphysema, chronic bronchitis, and respiratory irritation even at the per-million (ppm) level [3,4]. Hence, there is a pressing need for reliable methods to monitor atmospheric NO_2_ in real time with accuracy [5,6]. Traditional metal oxides are widely used in the gas sensing field due to their low cost, easy integration, excellent physicochemical properties, and unique structures [7,8]. In light of the drawbacks of high operating temperatures (>200 °C) and the limited sensitivity and slow response of metal oxide-based sensors, recent research has increasingly focused on nanoscale material design. Various nanostructures with a high surface-to-volume ratio, such as nanorods [9], nanofibers [10], nanotubes [11], and nanoflowers [12], have been widely employed in gas sensor fabrication to enhance sensing performance. Beyond these nanostructures, two-dimensional (2D) materials, composed of single or several atomic layers, are different from those based on conventional metal oxides [13], in which electrical dipoles are formed on the 2D surface by physisorption. Hence, 2D planar structures are considered suitable materials due to being highly sensitive [14], low power consuming, and uniquely selective to NO_2_ sensing [15].

Nevertheless, conventional metal oxides have a non-layered structure in two dimensions [16], which grants them the unique features of abundant surface dangling bonds and crystal structure distortion, thus improving their kinetic and gas sensing properties. However, metal oxides with a non-layered structure are usually difficult to prepare by traditional approaches. Although a variety of non-layered materials have been synthesized successfully in the 2D ultrathin form [17], such as TiO_2_ [18], In_2_O_3_ [19], and WO_3_ [20], utilizing diverse exfoliation techniques, achieving a uniform and large-area 2D structure remains difficult. Using the recently emerging liquid metal printing method, we can prepare large-area metal oxides and control the oxygen concentration. When the low-melting-point metal indium is heated to the liquid state, the surface metal will be oxidized to form an oxide film to prevent further reaction between the oxygen and the liquid metal internally, and then, through the van der Waals force, the oxide film will be reproduced [21]. For example, Cheng et al. [22] successfully synthesized large-area 2D In_2_O_3_ crystals by the liquid metal printing method, which displayed excellent response and selectivity for NO_2_ gas at room temperature with 365 nm light illumination.

Additionally, while conventional metal oxides have a wide band gap, containing insulators and semiconductors, they also have the defects of higher operating temperature, lower gas sensing response, poor gas selectivity, and difficulty in excitation of visible light due to their wide band gap [23,24]. To address these issues, we focused on ultrathin two-dimensional transition metal sulfides with special physicochemical properties and electronic properties at atomic level thicknesses. Metal sulfides have narrower and more-favorable bandgaps for visible light absorption, e.g., In_2_S_3_ has a bandgap of 1.9–2.5 eV, which is greatly reduced compared with that of In_2_O_3_ (3.0–3.8 eV). Using metal oxides to form heterojunctions with semiconductors (e.g., metal sulfides) with low-energy bandgaps effectively reduces the bandgap and is an effective way to achieve visible-light-driven room-temperature gas sensing performance [25,26]. Xu Kai [27] and others formed a unique oxygen sulfide heterostructure by doping the surrounding oxygen atoms into In_2_S_3_, which greatly improved the gas sensing performance of the material by increasing the exciton lifetime generated by photoexcitation by more than two orders of magnitude compared with that of pure In_2_S_3_. Chung Kim Nguyen et al. [28] reported on the 2D oxysulfide semiconductor, which was fabricated by printing indium oxide skins and a low-temperature wet chemical reaction; it exhibited a notably high electron mobility of ~20.4 cm^2^ V^−1^ s^−1^ and had excellent performance in the ultraviolet (UV) region.

To overcome these limitations, we turned to metal sulfides, which offer advantageous properties such as a small bandgap, room-temperature operation, and high selectivity. By constructing heterojunctions between metal oxides and these narrow-bandgap metal sulfides, we aim to develop high-performance gas sensors that operate at room temperature and are activatable by visible light. In this work, we prepare large-area, high-quality two-dimensional In_2_O_3_ by extracting the self-limited oxide layer from liquid indium droplets in a controlled environment and place the two-dimensional In_2_O_3_ under high-temperature conditions and a H_2_S atmosphere. The replacement reaction of O atoms and S atoms will occur on the surface of In_2_O_3_ to form a non-layered metal-oxygen sulfide two-dimensional transition state material In_2_S_x_O_3−x_. The electrical properties and gas sensing abilities of In_2_O_3_ and transition state material In_2_S_x_O_3−x_ are then studied to determine the effect of sulfidation on the precursor and the role of the gas sensing mechanism. Finally, we obtain a low-power, highly sensitive, highly stable room-temperature gas sensor with full recovery. We investigate the gas sensing application of transition state In_2_S_x_O_3−x_ formed by large-area metal oxides and construct a model of the gas sensing mechanism, which will provide guidance for the subsequent preparation of gas sensing sensor devices for two-dimensional non-laminated metal-oxygen sulfides.

## 2. Experimental Section

### 2.1. Synthesis of Two-Dimensional In_2_O_3_ and In_2_S_x_O_3−x_

The liquid metal van der Waals exfoliation synthesis process was performed under ambient conditions (20.9% O_2_, Shimao Gas Co., Ltd., Chengdu, China). Typically, the lateral dimension of the printed ultrathin layer is determined by the size of the indium liquid droplet. In this paper, 3 mg of the indium metal was utilized to obtain a sensitive layer of approximately 100 μm. Specifically, a heating plate was set to 180 °C to completely melt a small droplet of indium metal (99.99%, Jingrui Alloy Co., Ltd., Nangong, China). Then the pre-existing native dark oxide layer was swept off by a glass rod, thereby exposing the underlying fresh metal surface to oxygen. After 30 s, a clean and preheated SiO_2_ substrate was gently pressed upon the surface of the molten liquid metal. Immediately afterward, the heating plate was turned off to allow the molten metal to cool naturally. Subsequently, the SiO_2_ substrate with the attached metal was then ultrasonicated in alcohol for 10 min to achieve separation. Finally, the large-area, high-quality two-dimensional In_2_O_3_, which had formed on the fresh metal surface, was obtained on the target wafer. The sulfurization process is shown in Figure S1. Specifically, the two-dimensional In_2_O_3_ sample was put into the center of the tube furnace; then the furnace was purged with N_2_ at a flow rate of 50 sccm for 30 min to displace the air. Subsequently, the gas was switched to H_2_S (50 sccm) for another 30 min to purge the residual N_2_. Finally, the furnace temperature was raised to 300 °C and maintained for 120 min under the H_2_S atmosphere. After naturally cooling to room temperature, the vulcanized 2D In_2_S_x_O_3−x_ material was obtained.

### 2.2. Characterization

The lateral dimensions and thickness of 2D In_2_S_x_O_3−x_ were measured using AFM (Park NX10, Park Systems, Seongnam-si, South Korea). Raman spectra were examined by Raman spectroscopy (Raman, Lab-RAM HR Evolution, HORIBA, Paris, France) with a laser source of 532 nm. The valence band analysis and chemical structure analysis were performed on an X-ray photoelectron spectroscopy (XPS, Thermo Fisher Scientific, Waltham, MA, USA) ESCALAB Xi+ with an Al Kα source. The electronic band structure of the material was calculated by scanning tunneling microscopy (STS) using the STS module of an atomic force microscope (Park NX 10, Park Systems, Seomgnaam-si, South Korea) with a bias voltage of −2 ~ 2 V and a tunneling current setting of 0.5 nA. The electrical measurements of the materials were performed using a source meter (Keithley 2602B, Tektronix, Beaverton, OR, USA). The real-time drain current measurements were conducted at a fixed V_ds_ of −0.02, −0.2, −0.4, −0.6, −0.8, and −1 V.

### 2.3. Sensor Fabrication and Measurement

The sensitive devices were fabricated by depositing a pair of Au electrodes with 100 nm thickness on SiO_2_ substrates coated with either In_2_O_3_ or In_2_S_x_O_3−x_. The sensing measurements were conducted at room temperature under blue light (460 nm) illumination. A mass flow system controlled the inlet of target gases, using N_2_ as the balance gas. The gas flow introduced into the testing chamber was maintained at 200 standard cubic centimeters per minute (sccm) using a computer-controlled multichannel gas flow controller. Prior to data acquisition, N_2_ was purged through the chamber for 30 min to remove residual air and stabilize the sensor components. The current response of the sensors was measured with a Tektronix 2602B digital multimeter at a sampling rate of 1 Hz.

## 3. Result and Discussion

**Figure 1:**
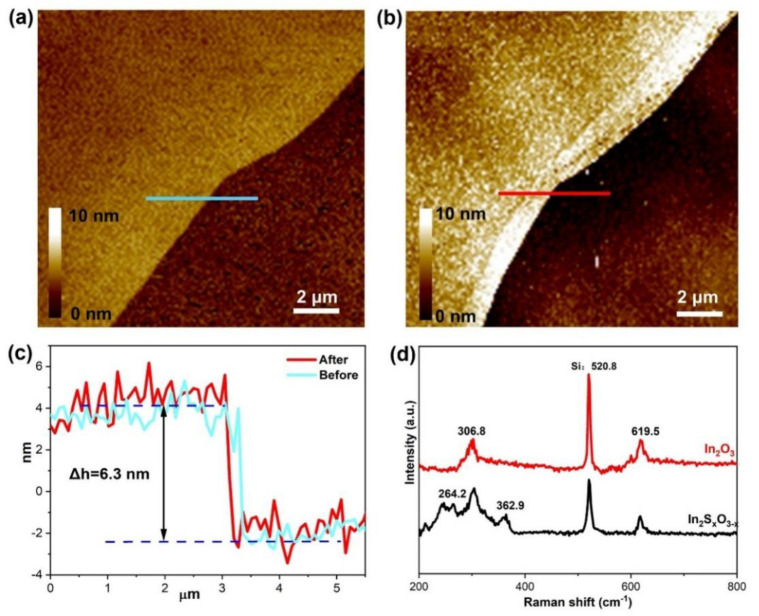
(**a**) AFM photographs of In_2_O_3_, with height calibrated by the blue line; (**b**) AFM photographs of In_2_S_x_O_3−x_, with height calibrated by the blue line; (**c**) AFM height maps containing blue (In_2_O_3_) and red (In_2_S_x_O_3−x_) lines; (**d**) Raman spectra of In_2_O_3_ and In_2_S_x_O_3−x_.

The 2D In_2_O_3_ nanosheets were synthesized via the liquid metal printing method. Subsequent sulfidation of these nanosheets through calcination treatment in H_2_S yielded 2D In_2_S_x_O_3−x_ nanosheets. As shown in Figure 1a,b, AFM measurements revealed the large-area morphology of both the synthesized In_2_O_3_ and In_2_S_x_O_3−x_. Profile analysis further confirmed that both In_2_O_3_ and In_2_S_x_O_3−x_ nanosheets exhibited a similar ultrathin thickness of ~ 6.3 nm with micrometer-scale lateral dimensions (Figure 1c). To investigate the chemical bond configurations, Raman spectroscopy was employed. As shown in Figure 1d, Raman peaks located at 520.8 cm^−1^ indicated Si from the background, which can be found in both samples. In addition, the peaks observed at 306.8 and 619.5 cm^−1^ are attributed to the vibrational modes of the In-O bond [6,29]. Upon sulfidation from In_2_O_3_ to In_2_S_x_O_3−x_, extra weaker shoulder peaks at 264.2 and 362.9 cm^−1^ arise from In–S vibrational modes [30,31], corresponding to the S atom’s replacement within the In_2_O_3_ structure in the calcination process.

**Figure 2:**
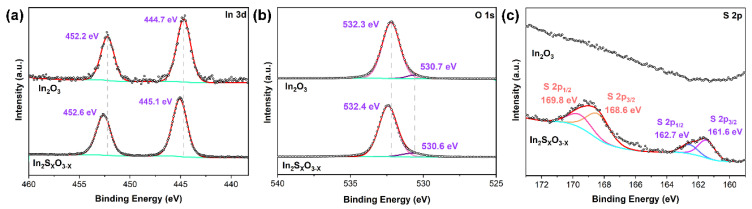
Black circles represent raw data and red lines are fitted envelopes. (**a**) High-resolution In 3d spectrum; (**b**) high-resolution O 1s spectrum; (**c**) high-resolution S 2p spectrum.The sulfurization of In_2_O_3_ induces a significant modification of the material’s electronic band structure. To quantify this change, STS measurements were conducted to determine the electronic band structure of 2D In_2_O_3_ and In_2_S_x_O_3−x_. To improve accuracy, we depicted five representative measurements and one STS result averaged from 40 *I–V* spectra shown as black circles in Figure 3. From Figure 3a, the STS spectrum of pristine In_2_O_3_ reveals a band gap of 3.08 eV. In contrast, the band gap of the In_2_S_x_O_3−x_ is reduced to 2.53 eV (Figure 3b), indicating a substantial narrowing. This narrowing is directly observed as a suppression of the tunneling current within the band gap region for both materials. According to the energy positions of the band edges, the valence band (VB) is located at approximately −1.98 eV for In_2_O_3_ and shifts to −1.50 eV for In_2_S_x_O_3−x_. Conversely, the conduction band (CB) shifts from +1.10 eV for In_2_O_3_ to +1.03 eV for In_2_S_x_O_3−x_. This upward shift of the VB is the primary contributor to the observed band gap reduction, suggesting a successful alteration of the electronic band structure through the partial replacement of oxygen with sulfur.

The surface compositions and chemical states of In_2_O_3_ and In_2_S_x_O_3−x_ were characterized by XPS. As shown in Figure 2a, two dominant peaks of In_2_O_3_ at 452.2 and 444.7 eV could be seen and were attributed to In 3d_3/2_ and In 3d_5/2_, respectively, representing In-O bonds [32]. Meanwhile, the In 3d spectra of In_2_S_x_O_3−x_ present two peaks at 452.6 and 445.1 eV, which show a slight blue-shift of 0.4 eV compared with In_2_O_3_. The shift in the binding energy of In in XPS is primarily attributed to changes in the atomic chemical environment, specifically the substitution of bonded atoms from oxygen (O) to sulfur (S), which alters the electron density around the indium atoms. As is well known, O has a high electronegativity, while S has a relatively lower electronegativity. In pristine In_2_O_3_, In is bonded to highly electronegative O atoms (forming In–O bonds). The oxygen atoms strongly attract the electron cloud from the outer shell of the indium atoms, resulting in a stronger effective binding of the core electrons to the nucleus. For In_2_S_X_O_3−X_, partial substitution of O atoms by S atoms leads to the formation of In–S bonds [33]. Due to the weaker electron-withdrawing ability of S atoms, the indium atoms bonded to sulfur retain a higher electron cloud density. The shift in the In 3d binding energy serves as the most direct and compelling spectroscopic evidence for the successful incorporation of sulfur, partial replacement of oxygen, and formation of In-S chemical bonds. In Figure 2b, the O 1s spectrum of In_2_S_x_O_3−x_ is similar to that of In_2_O_3_. The peaks at 532.3 eV in the O 1s spectrum are attributed to Si-O bonds that are associated with the sample background, and the shoulder peak at 530.7 eV is associated with the formation of In-O bonds [6]. However, as shown in Figure 2c, the S 2p spectrum of In_2_S_x_O_3−x_ reveals distinct peaks at 169.8 eV and 169.1 eV, as well as at 162.7 eV and 161.8 eV, which can be, respectively, assigned to the S 2p_1/2_ and S 2P_3/2_ components of S-O [34] bonds and to the S 2p_1/2_ and S 2p_3/2_ components of In-S bonds [35]. The formation of O-S bonds withdraws electron density from the In atoms. This electron withdrawal reduces the charge density at adjacent metal sites, effectively increasing their oxidation state and accounting for the observed positive shift (~0.4 eV) in the In 3d binding energy. The formation of In-S bonds suggests that elevated temperature weakens the original In-O bonds, leading to cleavage of the crystal structure of indium oxide and allowing oxygen atoms to be replaced by sulfur atoms through vulcanization. From Figure S2, the composition of the obtained 2D In_2_S_X_O_3−X_ can be estimated to be In: S: O = 2:0.35:2.62 by using the O:S area ratio obtained from XPS. The result indicates the value of x in In_2_S_x_O_3−x_ as ~0.35.

**Figure 3:**
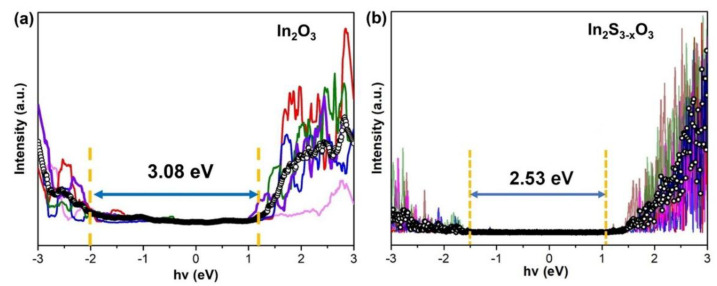
The colored lines represent five representative measurements and the black circles is the STS result averaged from 40 I−V spectra of (**a**) In_2_O_3_ and (**b**) In_2_S_x_O_3−x_.

**Figure 4:**
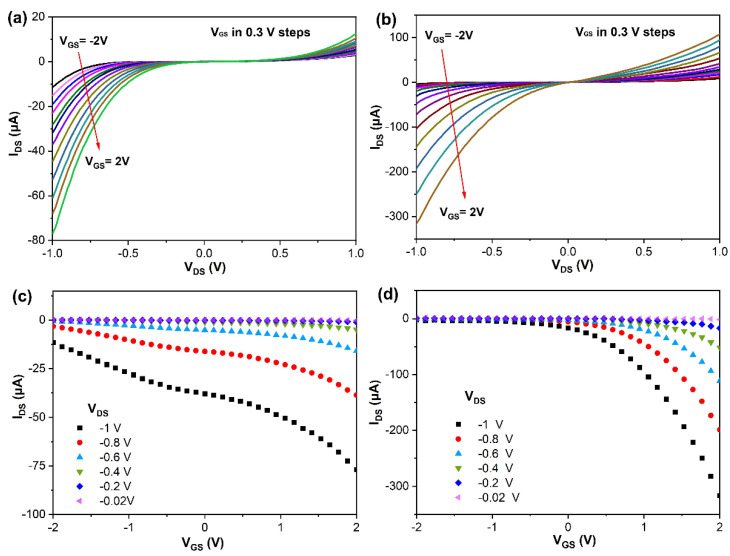
Output *I*_DS_–*V*_DS_ characteristics of (**a**) In_2_O_3_, (**b**) In_2_S_x_O_3−x_; gate voltage and drain current transfer curves of the devices based on (**c**) In_2_O_3_, (**d**) In_2_S_x_O_3−x_.

To further characterize their electrical properties, the output drain source current (*I*_DS_) with respect to variation of the drain source voltage *V*_DS_ (−1.0 to 1.0 V) is illustrated in Figure 4a,b for In_2_O_3_ and In_2_S_x_O_3−x_, respectively. The resulting curves evaluate the viability of these materials for photodetection applications. The analysis reveals that In_2_S_x_O_3−x_ supports a higher current than In_2_O_3_ at the same bias voltage. This enhanced current offers practical advantages, including greater signal strength, improved anti-interference capability, a wider dynamic range, and better circuit compatibility [36,37]. Furthermore, the transfer characteristics *I*_DS_ with respect to variation of back-gate voltage (*V*_GS_) of the fabricated In_2_O_3_ and In_2_S_x_O_3−x_ devices are shown in Figure 4c,d, which exhibit that both materials are n-type semiconducting materials. The mobility of 2D In_2_O_3_ and In_2_S_x_O_3−x_ devices was calculated from the linear segment of the *I*_DS_–*V*_GS_ curve elucidated via the following equation

$$\mu =\left(\frac{∆I_{DS}}{∆V_{GS}}\right)\times \frac{L}{\left(WCV_{DS}\right)}$$

where *L*, *W*, and *C* are the channel length, channel width, and gate capacitance per unit area, respectively. The *L* and *W* are 20 and 40 μm for all devices, respectively. The capacitance per unit area between the channel and the back gate (Cox) was calculated to be 11.5 × 10^−9^ F cm^−2^ for the 300 nm dielectric SiO_2_ layer. The calculated mobility of the In_2_S_x_O_3−x_ device is ~11.1 cm^2^ V^−1^ s^−1^ at *V*_DS_ = 1 V, which is higher than the value for In_2_O_3_ (4.10 cm^2^ V^−1^ s^−1^). These results further demonstrate the superior electrical properties of In_2_S_x_O_3−x_ and denote that it is more appropriate for the photodetector and resistive gas sensor.

**Figure 5:**
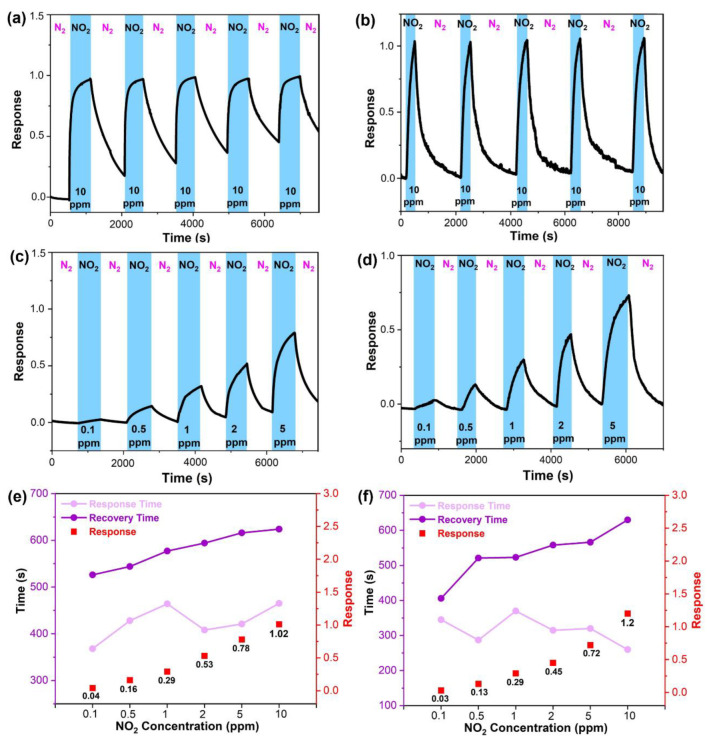
The dynamic response curves of (**a**) In_2_O_3_; (**b**) In_2_S_x_O_3−x_ upon the exposure to 10 ppm NO_2_ gas in 5 repeated cycles; the dynamic response curves of (**c**) In_2_O_3_; (**d**) In_2_S_x_O_3−x_ sensor upon the exposure to NO_2_ gas with different concentrations at room temperature under blue light (460 nm); the response time and recovery time of (**e**) In_2_O_3_; (**f**) In_2_S_x_O_3−x_.

To experimentally evaluate the NO_2_ sensing performance of 2D In_2_S_x_O_3−x_, sensors were fabricated by depositing a pair of Au electrodes with 100 nm thickness on SiO_2_ substrates coated with either In_2_O_3_ or In_2_S_x_O_3−x_. The sensing measurements were conducted at room temperature under blue light (460 nm) illumination. A mass flow system controlled the inlet of target gases, using N_2_ as the balance gas. The sensing response was evaluated by the calculation formula $\frac{I_{N_{2}}-I_{g}}{I_{g}}$, where I_g_ is the sensor current in the analyte gas and I_N2_ is the current in pure N_2_. Figure 5a shows the response of the In_2_O_3_ sensor for five repeated cycles with 10 ppm NO_2_. It exhibited a response factor of 1.02, along with degrading recovery and a shifting baseline over successive cycles. In contrast, the In_2_S_x_O_3−x_ sensor demonstrated superior performance under the same conditions (Figure 5b), with a higher response factor of 1.2, better recovery performance, and significantly improved stability over five cycles. The dynamic response to NO_2_ concentrations ranging from 0.1 to 5 ppm was also investigated. The In_2_O_3_ sensor yielded response factors of 0.04, 0.16, 0.29, 0.53, and 0.78, as shown in Figure 5c. The In_2_S_x_O_3−x_ sensor showed comparable response factors of 0.03, 0.13, 0.29, 0.45, and 0.72, as shown in Figure 5d, indicating a similar sensitivity to In_2_O_3_ in this concentration range. A key difference was observed in response time. The response time, the time to reach 90% of the maximum response, for In_2_S_x_O_3−x_ was significantly shorter than for In_2_O_3_, especially at higher gas concentrations as shown in Figure 5e,f. While their response times were similar at low NO_2_ levels, In_2_S_x_O_3−x_ responded much faster as the concentration increased, with times of 260 s and 465 s for In_2_S_x_O_3−x_ and In_2_O_3_, respectively, at 10 ppm. In addition, the recovery time, which is defined as the time to recover to 10% of the maximum response, for both sensors followed a similar trend that increased with NO_2_ concentration. Selectivity was assessed by exposing the sensors to various industrial gases, including 3% H_2_, 300 ppm NH_3_, 500 ppm CO_2_, and 50 ppm SO_2_. As shown in Figure S3, both In_2_O_3_ and In_2_S_x_O_3−x_ exhibit a relatively better selectivity to NO_2_ than to any other gas, despite NO_2_ being tested at a concentration at least 5-fold lower. Additionally, through a linear fit of response versus concentration and a fifth-order polynomial fit to the noise (Figure S4, Table S1), the sensor demonstrates an excellent theoretical detection limit of 31.3 ppb. Furthermore, the NO_2_ sensing performance of In_2_S_x_O_3−x_ is benchmarked against other reported In_2_O_3_ and In_2_S_3_ sensors in Table 1. As we all know, an ideal gas sensor requires high response and selectivity, low operating temperature, and a low detection limit. The In_2_S_x_O_3−x_ sensor demonstrates a comparatively high response to low-concentration NO_2_ at room temperature, offering a simple path toward potentially lower-cost sensors.

**Figure 6:**
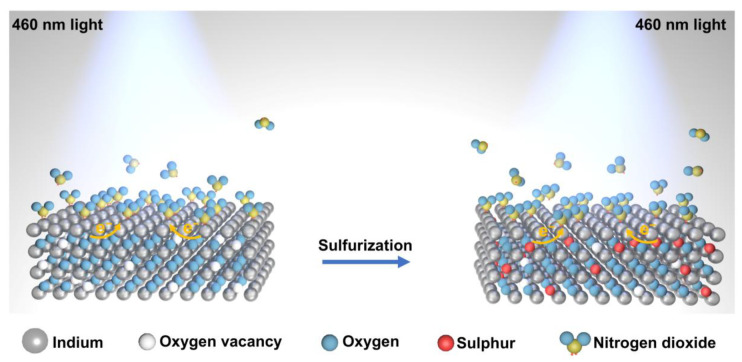
Schematic diagram of In_2_O_3_ sulfurto In_2_S_x_O_3−x_ process and gas sensing mechanism diagram.

Unlike conventional metal oxide-based sensors, the interaction between NO_2_ and this 2D surface is majorly dominated by physisorption. The fully reversible and stable resistance cycles of In_2_S_x_O_3−x_ (Figure 5b), with minimal baseline drift, are characteristic of predominant physisorption. This behavior contrasts with the baseline drift often associated with strong chemisorption or surface oxidation. This mechanism enables operation at room temperature, significantly reducing power consumption [45,46]. We propose that the sulfurization process, through the replacement of oxygen with sulfur, increases the carrier concentration of the material. Consequently, under identical test conditions, In_2_S_x_O_3−x_ exhibits better recovery performance than In_2_O_3_. Furthermore, the optical driving method can excite electrons and induce them to migrate to the surface of the material, further enhancing the carrier concentration at the surface. This reduces the required excitation energy, allowing In_2_S_x_O_3−x_ to be activated by visible light of longer wavelengths than required for In_2_O_3_, as shown in the schematic diagram of Figure 6. When NO_2_ molecules contact the material, electron dipoles are formed on the interface between In_2_S_x_O_3−x_ and NO_2_ due to its paramagnetic nature. This promotes charge transfer from the n-type In_2_S_x_O_3−x_, which acts as an electron donor, to the electron-accepting NO_2_ molecules. The consequent decrease in the carrier concentration of the material leads to an increase in resistance and resistance recovery after the desorption of NO_2_ [47].

## 4. Conclusions

A large lateral dimension of the 2D In_2_S_x_O_3−x_ nanosheet in micrometer scale was synthesized using a liquid metal printing method for In_2_O_3_ followed by a controlled annealing treatment. This annealing process induced sulfurization, partially replacing oxygen with sulfur to transform In_2_O_3_ into In_2_S_x_O_3−x_. The resulting material demonstrated highly selective and completely reversible NO_2_ sensing performance at room temperature. Individual 2D flakes of both In_2_O_3_ and In_2_S_x_O_3−x_ were isolated with thicknesses of ~6.3 nm. Sulfurization significantly modified the bandgap, yielding an n-type semiconducting material. The electronic structure of 2D In_2_S_x_O_3−x_ was particularly favorable for NO_2_ physisorption and interfacial charge transfer due to optimal band alignment and carrier concentrations. This was supported by I–V characterization, which revealed the superior electrical properties of In_2_S_x_O_3−x_ compared with In_2_O_3_. Consequently, the In_2_S_x_O_3−x_ sensor demonstrated excellent performance at room temperature, achieving response factors from 0.03 to 1.2 for low NO_2_ concentrations (0.1 to 10 ppm) under 460 nm visible light excitation. The sensor was highly selective towards NO_2_ despite being tested at concentrations at least five times lower than those of other referential gases. It also exhibited complete reversibility and a faster response time. This study establishes 2D non-layered metal oxysulfides as a promising class of high-performance sensing materials with strong potential for developing lower-cost, low-power, and room-temperature NO_2_ sensors.

## Figures and Tables

**Table 1:** Performance comparison of the NO_2_ sensors based on In_2_S_3_ or In_2_O_3_ materials.

Materials	Temperature (°C)	Light Cond.	NO_2_ (ppm)	Response $\frac{R_{g}-R_{a}}{R_{g}}$	t_res._/t_rec_ (s)	LOD (ppb)	Reference
In_2_O_3_ microflowers	100	Dark	1	62.6	344/318	—	[38]
In_2_O_3_ nanocubes	50	Dark	3	9.0	21/522	60.0	[39]
In_2_O_3_ nanospheres	120	Dark	1	370.9	148/72	10.0	[40]
In_2_O_3_/SnS_2_ nanoflowers	180	Dark	5	14.6	34/65	12.0	[12]
In_2_O_3_@WO_3_ nanowires	200	Dark	3	7.4	22.2/216	—	[41]
As-doped In_2_O_3_ nanowires	RT	Dark	500	0.7	—/—	500.0	[42]
2D/2D MoS_2_/In_2_S_3_	RT	Dark	50	1.9	96/408	450.0	[43]
Au@In_2_S_3_/In_2_O_3_ hybrid microflowers	RT	Dark	100	19.7	12/27	200.0	[44]
In_2_S_3−x_O_3_	RT	460 nm	10	1.2	260/620	31.3	This work

Temperature.: working temperature; RT: room temperature; Light cond.: Light condition; $R_{g}$: resistance of the sensor exposed to NO_2_; $R_{a}$: resistance of the sensor exposed to air or N_2_; t_res._: response time; t_rec._: recovery time; LOD: low detection limit.

## Data Availability

Data are contained within the article.

## Supplementary Materials

The following supporting information can be downloaded at: https://www.mdpi.com/article/10.3390/s26020670/s1, Figure S1: (a) Tube furnace, (b) Schematic diagram of vulcanization process; Figure S2. The integral areas of (a) O 1s and (b) S 2p peaks from the O 1s XPS spectra of In_2_S_X_O_3−x_; Figure S3. Selectivity tests of 3% H_2_, 300 ppm NH_3_, 500 CO_2_, 50 ppm SO_2_ and 10 ppm NO_2_ for In_2_O_3_ and In_2_S_x_O_3−x_; Figure S4 (a) Response at different NO_2_ concentrations (black dots) and the corresponding fit curve. (b) The 5th order polynomial fitted response of the sensor versus time at the baseline before NO_2_ exposure; Table S1: 5th order polynomial fitting data for sensor.
