# Supplementary material for: Enhanced Room-Temperature Optoelectronic NO2 Sensing Performance of Ultrathin Non-Layered Indium Oxysulfide via In Situ Sulfurization
Source: Sensors (Basel). 2026 Jan 19;26(2):670. doi: 10.3390/s26020670 (PMC12845805; doi:10.3390/s26020670)
Supplement: Supplementary file 1 [file sensors-26-00670-s001.zip › sensors-4067536-supplementary.pdf]

Supporting Information

Enhanced Room Temperature Optoelectronic NO_2 Sensing Performance of Ultrathin Nonlayered Indium Oxysulfide via In-situ Sulfurization

Yinfen Cheng[1], Nianzhong Ma[2], Zhong Li[2], Dengwen Hu[2] Zhentao Ji[2], Lieqi Liu[2], Rui Ou[3], Zhikang Shen[4], Jian Zhen Ou[2, 3, *]

[1]Institute for Advanced Study, Chengdu University, Chengdu, 610106, China; chengyinfen@cdu.edu.cn

[2]Key Laboratory of Advanced Technologies of Materials, Ministry of Education, School of Materials Science and Engineering, Southwest Jiaotong University, Chengdu 610031, China; nzma@my.swjtu.edu.cn (N.M.); zhong.li@swjtu.edu.cn (Z.L.); hudw@swjtu.edu.cn (D.H.); difuling@my.swjtu.edu.cn (Z.J.); llq@my.swjtu.edu.cn (L.L.)

[3]School of Engineering, Royal Melbourne Institute of Technology University, Melbourne 3000, Australia; rui.ou@ucas.ac.cn

[4]College of Engineering and Technology, Southwest University, Chongqing, 400715, China; zhikangshen@outlook.com

*Corresponding author. E-mail: jianzhen.ou@rmit.edu.au .

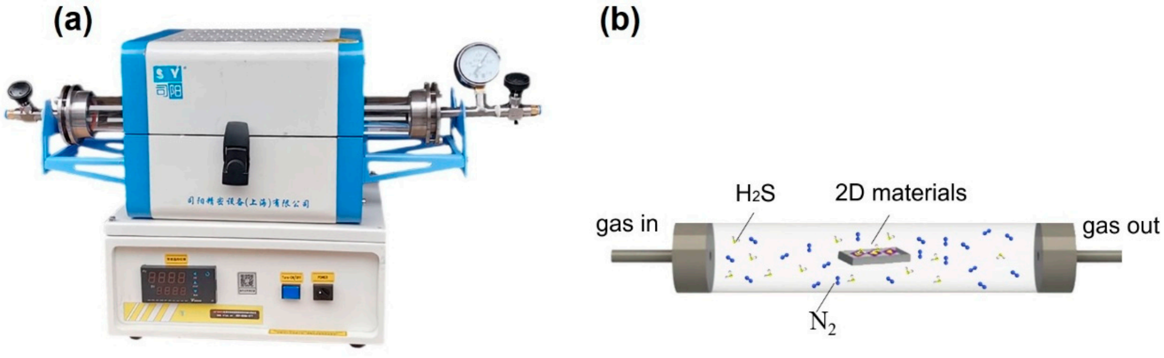

Figure S1. (a) Tube furnace, (b) Schematic diagram of vulcanization process

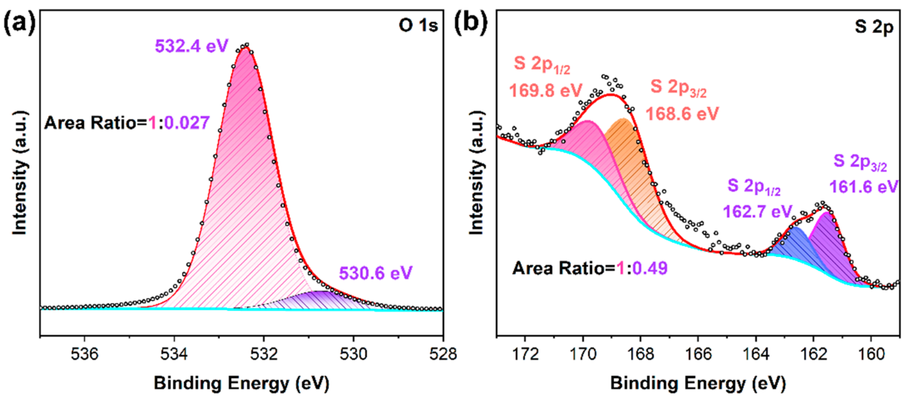

Figure S2. The integral areas of (a) O 1s and (b) S 2p peaks from the O 1s XPS spectra of $In_2S_XO_{3-X}$.

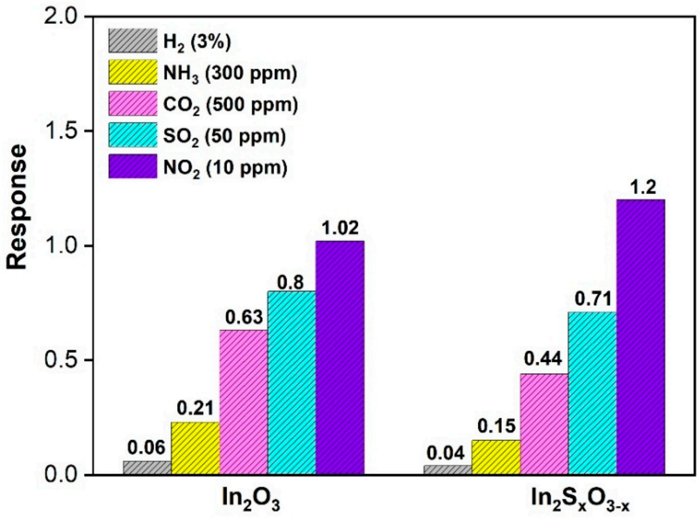

Figure S3. Selectivity tests of 3% H_2, 300 ppm NH_3, 500 CO_2, 50 ppm SO_2 and 10 ppm NO_2 for In_2O_3 and $In_2S_xO_{3-x}$

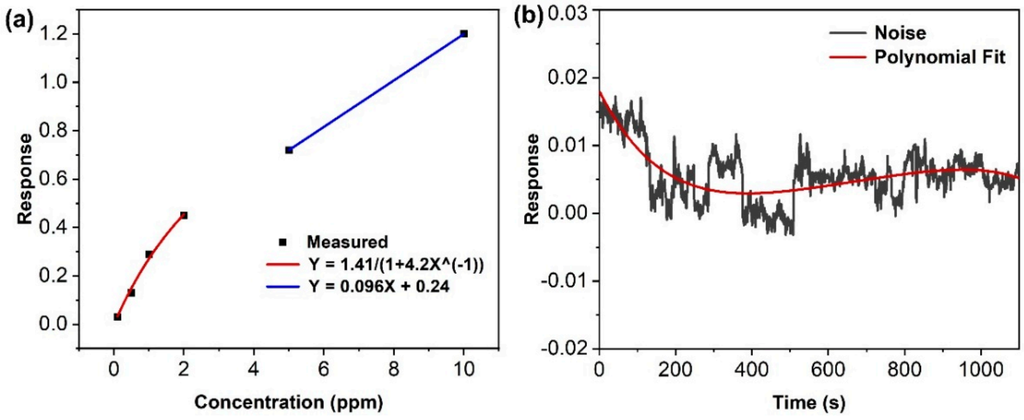

Figure S4 (a) Response at different NO_2 concentrations (black dots) and the corresponding fit curve. (b) The 5th order polynomial fitted response of the sensor versus time at the baseline before NO_2 exposure.

Table S1 5th order polynomial fitting data for sensor

Time (s)	Yi-Y	(Yi-Y)^2
1	-0.003498	1.2236E-05
100	0.003095	9.57903E-06
200	0.000157	2.4649E-08
300	0.006496	4.2198E-05
400	-0.00475	2.25625E-05
500	-0.00519	2.69361E-05
600	0.001067	1.13849E-06
700	-0.000047	2.209E-09
800	0.001805	3.25803E-06
900	-0.001746	3.04852E-06
1000	0.000674	4.54276E-07

$$V_x^2 = \sum (Y_i - Y)^2 = 0.000121$$

$$RMS(ppm^{-1}) = \sqrt{(V_x^2/(N-1))} = 0.00348$$

$$Lod(ppm^{-1}) = 0.0313\,ppm = 31.3\,ppb$$

Here, Y_i denotes the response value measured at a given time point after the sensor has reached a stable state in a nitrogen atmosphere. At the same time, Y represents the corresponding value obtained from a fifth-order polynomial fitting. The noise level, expressed as the root-mean-square (RMS) deviation, quantifies the baseline fluctuation of the sensor under steady conditions. The limit of detection (LOD) is the minimum concentration of the target gas that yields a sensor response equal to 3 times the steady-state noise level.
